# Familial left ventricular noncompaction cardiomyopathy associated with the p.Asp461Asn *MYH7* variant

**DOI:** 10.1515/biol-2025-1308

**Published:** 2026-03-20

**Authors:** Yanyan Zhou, Yuqi Wang, Xiaoqing Yang, Jie Mi, Fang Liang

**Affiliations:** Department of Geriatrics, Shijiazhuang People’s Hospital, Shijiazhuang, 05000, China; Department of Radiology, Shijiazhuang People’s Hospital, Shijiazhuang, 05000, China; Department of Cardiology, Shijiazhuang People’s Hospital, Shijiazhuang, 05000, China

**Keywords:** familial, gene variant, *MYH7* gene, noncompaction cardiomyopathy

## Abstract

Left ventricular noncompaction cardiomyopathy (LVNC) is a distinct form of cardiomyopathy that may present as either an inherited or a sporadic condition. This report describes the first documented case of familial LVNC associated with the *MYH7* p.Asp461Asn variant. Phenotypic variability among affected individuals within the family was assessed to identify potential contributors to the observed clinical heterogeneity in LVNC. The reported family included 10 individuals across three generations. Two members were diagnosed with LVNC, and 1 was classified as having suspected LVNC. Identical twins (Ⅱ-1 and Ⅱ-3) were both found to harbor the heterozygous missense variant c.1381G > A (p.Asp461Asn) in the *MYH7* gene. Subsequent pedigree analysis confirmed the presence of this variant in individuals Ⅲ-1, Ⅲ-2, and Ⅲ-4. Clinical observations from this familial case highlight the importance of early identification and intervention in patients with LVNC to mitigate the risks of heart failure, sudden cardiac death, and thromboembolic events. The *MYH7* variant plays a significant role in the pathogenesis of LVNC and may represent a promising target for future gene-based therapies aimed at improving patient outcomes.

## 1 Introduction

Left ventricular noncompaction cardiomyopathy (LVNC) is a form of inherited or sporadic cardiomyopathy characterized pathologically by prominent myocardial trabeculations and deep intertrabecular recesses. Common clinical presentations include biventricular dysfunction or isolated right ventricular involvement. The pathogenesis of LVNC remains incompletely understood and is not attributable to a single genetic factor. Based on existing evidence, variants in the *G4.5* gene (located on chromosome Xq28) have been shown to contribute to disease onset in pediatric populations, whereas abnormalities in the autosomal 11p15 region are more frequently associated with adult cases. Additional proposed contributing factors include dysregulated activity of tumor necrosis factor-alpha converting enzyme, subendocardial myocardial hypoxia, and various teratogenic influences.

The clinical manifestations of LVNC are heterogeneous and often non-specific, encompassing heart failure, arrhythmias, thromboembolic events [1], 2], or asymptomatic presentations. Symptom severity may range from the absence of clinical signs to advanced heart failure, arrhythmias, thromboembolic complications, and sudden cardiac death (SCD), electrocardiogram abnormalities are commonly present [3]. Studies have reported that malignant arrhythmias and SCD account for 2–5 % of cases.

LVNC may occur as part of a syndromic presentation (e.g., Barth syndrome), as a congenital heart defect (e.g., Ebstein anomaly), or as an isolated familial cardiomyopathy. A familial history is reported in approximately 18–24 % of cases. The prevalence of the condition is higher among men than among women [4]. This report presents a three-generation pedigree of a family affected by noncompaction cardiomyopathy (NCCM) associated with the missense variant c.1381G > A (p.Asp461Asn) in the myosin heavy chain 7 gene (*MYH7*), a variant previously reported only in cases of dilated cardiomyopathy [5]. The phenotypic variability among affected family members is also described to explore potential contributors to the clinical heterogeneity observed in LVNC, with the aim of improving awareness and understanding of the disease.

## 2 Case report

### 2.1 Case data

**Figure 1:**
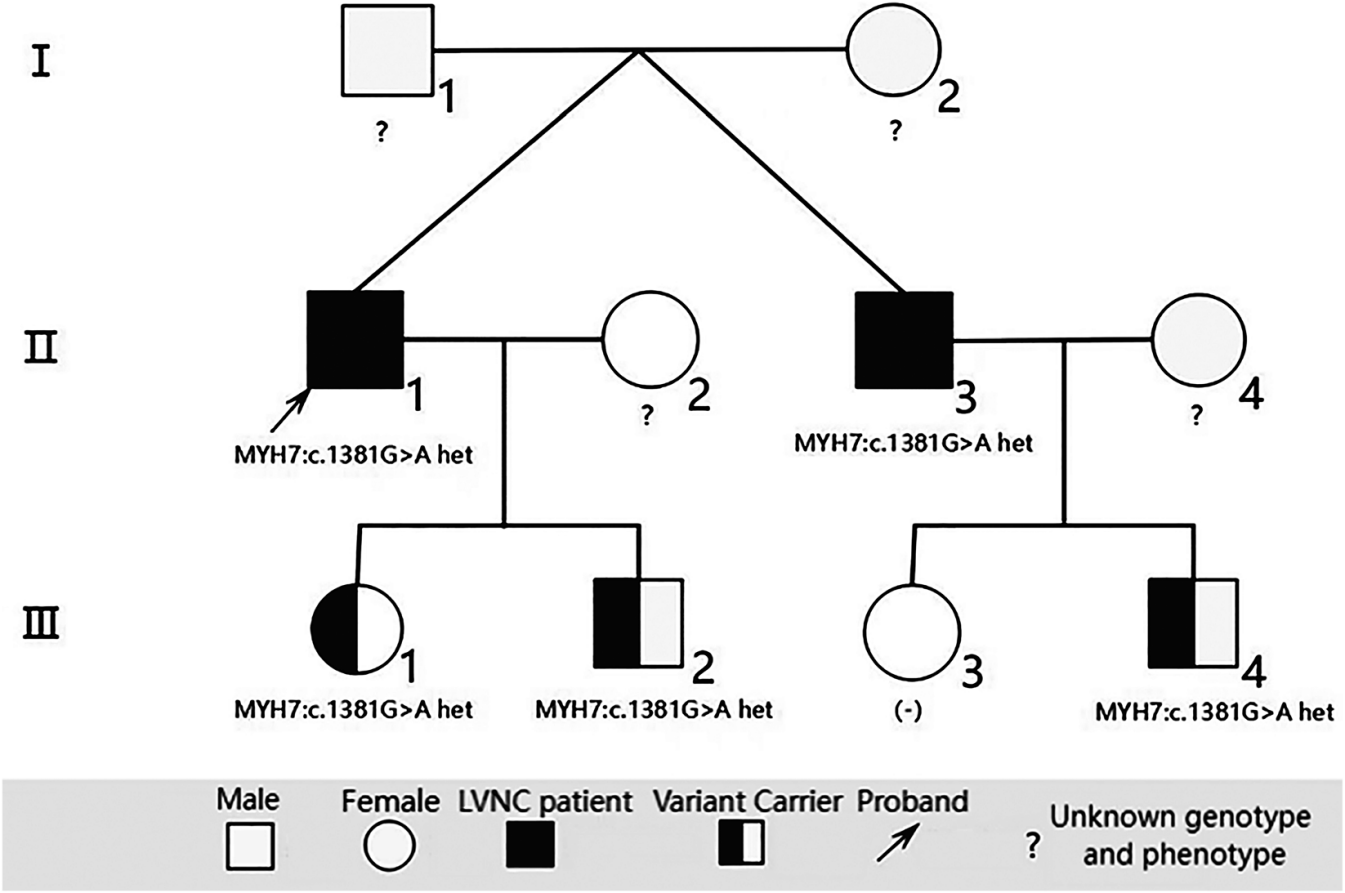
Three-generation pedigree of a family with left ventricular noncompaction cardiomyopathy segregating the MYH7 p.Asp461Asn variant. Filled symbols indicate confirmed LVNC (individuals II-1 and II-3), half-filled denotes suspected LVNC (III-4), and unfilled represents unaffected status. Genotype status is shown below each tested member, with carriers including II-1, II-3, III-1, III-2, and III-4, demonstrating autosomal dominant inheritance with incomplete penetrance.

Pedigree investigation: The pedigree investigation was initiated following the provision of informed consent by the patients and their family members. The diagnostic criteria for NCCM were based on the echocardiographic Jenni criteria. The family comprised 10 members across 3 generations (Figure 1), among whom 2 were diagnosed with LVNC and 1 was classified as having suspected LVNC. Notably, individuals Ⅱ-1 and Ⅱ-3 are identical twin brothers.

Individual Ⅱ-1: This 48-year- old male was admitted to the hospital on May 17, 2023, with a complaint of “increased narcolepsy for 4 years, worsening for 1 day.” Four years earlier, episodes of sudden, uncontrollable sleepiness occurred without an identifiable cause. Brief arousals for eating and defecation were reported, followed by rapid return to sleep. These episodes lasted for two consecutive days and resolved without residual symptoms. Intermittent recurrences were noted approximately twice, each lasting 2–3-days, and occasionally accompanied by lockjaw and urinary incontinence, both of which resolved spontaneously. Three years prior, a medical evaluation at a local hospital was sought due to prolonged sleep duration, unresponsiveness, and lethargy. A tentative diagnosis of metabolic encephalopathy secondary to ketosis was suggested. One day prior to admission, excessive sleepiness recurred without an obvious cause. The patient could be aroused by external stimulation but quickly returned to sleep, without associated fecal or urinary incontinence.

**Figure 2:**
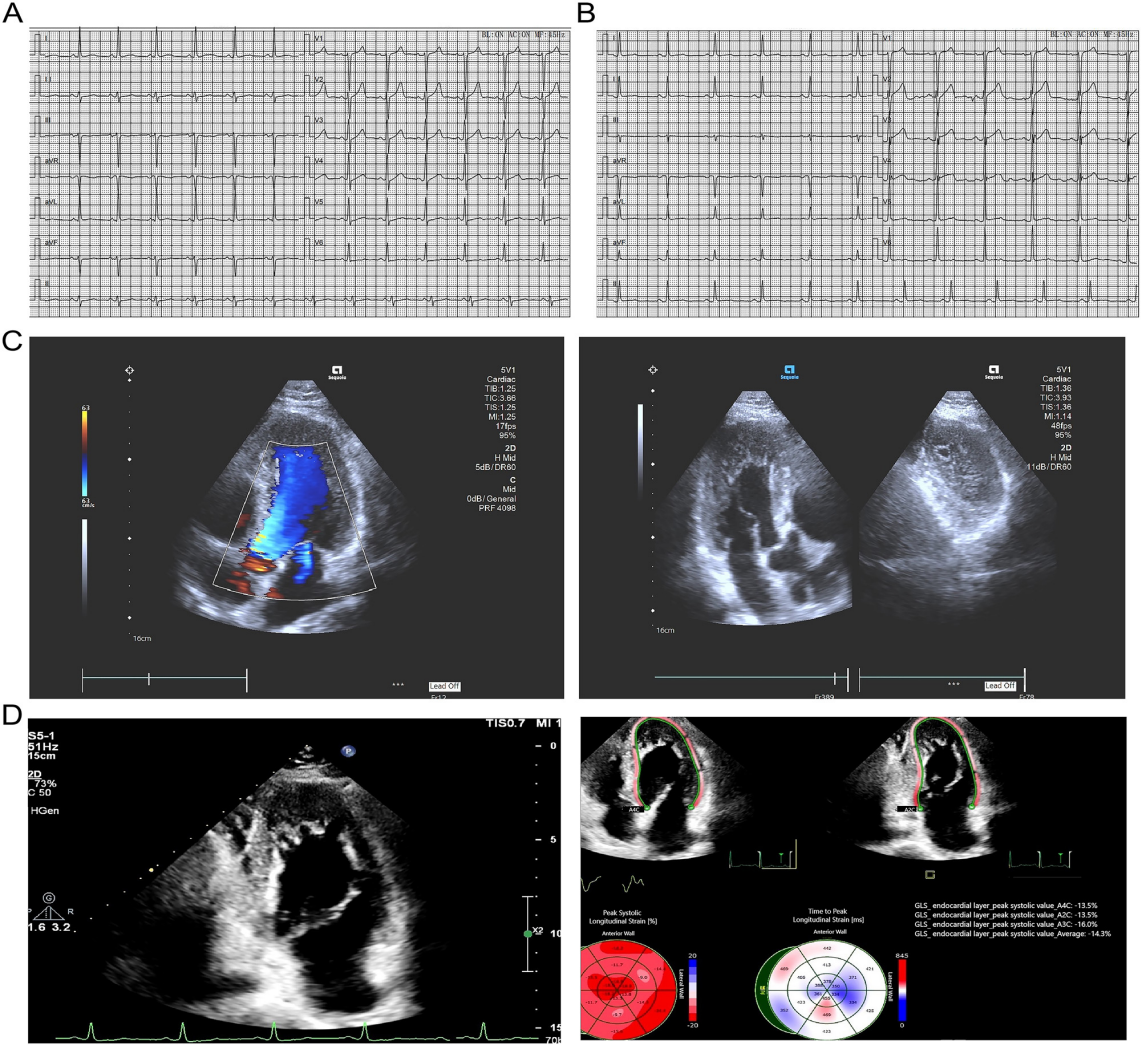
ECG and TTE findings in individuals Ⅱ-1 and Ⅱ-3. A: ECG of Ⅱ-1 presenting sinus rhythm with T-wave abnormalities. Paper speed: 25 mm/s sensitivity: 10 mm/mM. B: ECG of Ⅱ-3 indicating sinus rhythm, left ventricular hypertrophy, and T-wave flattening. Paper speed: 25 mm/s sensitivity: 10 mm/mM. C: TTE of Ⅱ-1 demonstrating left ventricular apical hypertrophy consistent with apical LVNC, mild aortic valve regurgitation, mild bicuspid and tricuspid valve regurgitation, and preserved left ventricular systolic function. D: TTE of Ⅱ-3 indicating features similar to LVNC and suspected right ventricular NCCM, mild bicuspid and tricuspid valve regurgitation, preserved left ventricular function, and reduced longitudinal strain in both left and right ventricular long axes.

Upon emergency evaluation, head CT revealed an old infarction in the right basal ganglia and thalamus. Laboratory findings included glucose 7.7 mmol/L, HbA1c 8.70 %, HbA1 9.90 %, urine acetone bodies 2+, urine glucose 4+. Arterial blood gas analysis showed PCO_2_ 33 mmHg, PCO_2_(T) 31.9 mmHg, total CO_2_ 22.4 mmol/L, AaDO_2_ 22.3 mmHg, Cl^−^ 107 mmol/L, and anion gap 17.3 mmol/L. ECG demonstrated sinus rhythm with T wave abnormalities. Transthoracic echocardiography (TTE) indicated left ventricular apical hypertrophy with morphological features consistent with apical LVNC, mild aortic regurgitation, mild bicuspid and tricuspid regurgitation, and preserved left ventricular systolic function (Figure 2). Whole-exome sequencing identified a heterozygous missense variant c.1381G > A (*MYH7*:p.Asp461Asn het), associated with hypertrophic cardiomyopathy type 1 (HCM1), and a heterozygous missense variant c.9094T > G (*RYR2*:p.Cys3032Gly het), considered a suspected pathogenic variant.

Individual Ⅱ-3: This 48-year-old male presented on May 12, 2023, for evaluation of elevated blood pressure persisting for eight months, with poor control during the preceding week. Eight months earlier, he experienced dizziness without an identifiable cause, and the highest recorded blood pressure was 160/110 mmHg. No symptoms of headache, chest distress, palpitations, or dyspnea were reported. Antihypertensive treatment with nifedipine sustained release tablets (10 mg, once daily, orally) was initiated at a local clinic, resulting in blood pressure control around 120/80 mmHg. One week prior to presentation, dizziness recurred, and blood pressure was 160/100 mmHg. Oral antihypertensive medication was administered, leading to temporary normalization of blood pressure and resolution of dizziness; however, blood pressure remained sub optimally controlled, fluctuating between 140 and 160/90–100 mmHg.

ECG indicated sinus rhythm and left ventricular hypertrophy with T wave changes. Coronary angiography revealed a right-dominant coronary circulation, no significant stenosis in the left main artery, diffuse stenosis (75–80 %) in the proximal and mid-segments of the left anterior descending artery, and scattered plaques in the circumflex and right coronary arteries (Figure 2). TTE demonstrated interventricular septal and apical myocardial thickening, reduced global left ventricular wall motion, increased apical trabeculation, and decreased systolic and diastolic function, with an ejection fraction of 48 %. Laboratory findings included fasting blood glucose 9.0 mmol/L, triglycerides 3.51 mmol/L, and Low-Density Lipoprotein Cholesterol (LDL-C) 4.35 mmol/L. Whole-exome sequencing identified the heterozygous missense variant c.1381G > A (*MYH7*:p.Asp461Asn het) associated with HCM1, and the heterozygous missense variant c.9094T > G (*RYR2*:p.Cys3032Gly het), suspected to be pathogenic.

Individuals Ⅲ-1 and Ⅲ-2: These individuals reported no clinical symptoms and declined further evaluation.

**Figure 3:**
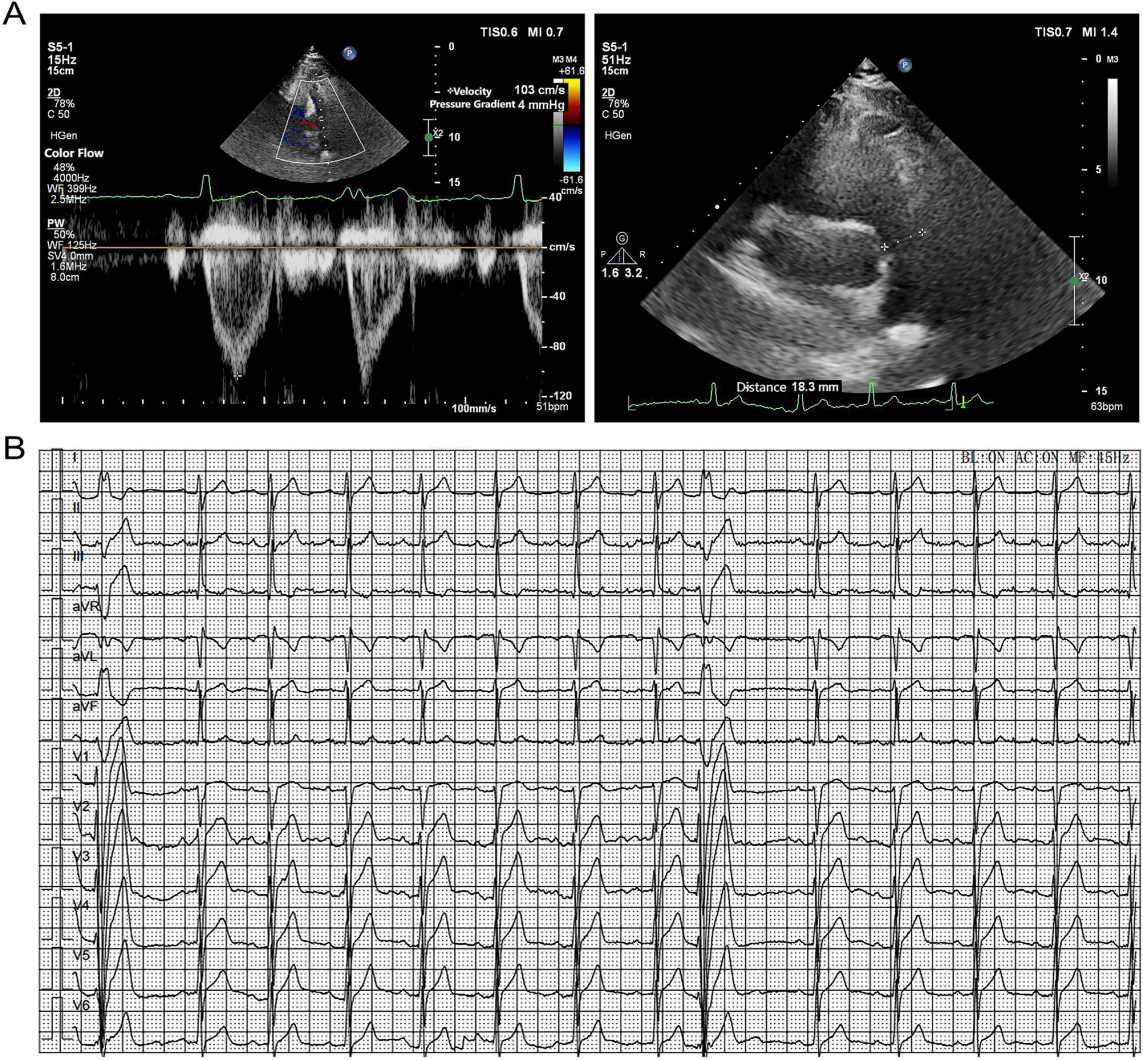
ECG and TTE findings in individual Ⅲ-4. A: UCG showing blood-filled sinusoids in the apical segment of the posterior and lateral walls of the left ventricle, with asynergic wall motion. B: ECG demonstrating sinus rhythm with PVCs. Paper speed: 25 mm/s sensitivity: 10 mm/mM.

Individual Ⅲ-4: This 19-year-old male underwent TTE, which demonstrated a left ventricular ejection fraction of 61 %. Blood-filled sinusoids were observed in the apical region of the posterior and lateral walls of the left ventricle. Left ventricular wall motion abnormalities were identified, considered secondary to premature ventricular contractions (PVCs). Overall left ventricular function was preserved. ECG findings revealed sinus rhythm and frequent PVCs. Left ventricular contrast echocardiography showed increased trabeculations in the inferior, posterior, and lateral walls of the left ventricle, with mildly reduced contractility (Figure 3). Laboratory results indicated LDL-C 3.71 mmol/L and total cholesterol 5.43 mmol/L. A diagnosis of frequent PVCs was established.

**Informed consent:** Informed consent has been obtained from all individuals included in this study.

**Ethical approval:** The research related to human use has been complied with all the relevant national regulations, institutional policies and in accordance with the tenets of the Helsinki Declaration, and has been approved by the Ethics Committee of the Shijiazhuang People’s Hospital (Approval Number: 2024-156).

### 2.2 DNA extraction

Following the provision of informed consent by the study participants, genomic DNA was extracted from venous blood samples using the QIAamp Blood DNA Extraction Kit (Qiagen, Germany). The absorbance value and concentration of the extracted DNA were subsequently measured.

### 2.3 NGS sequencing

Next-generation sequencing was used for genetic analysis. Initially, 1 μg of extracted DNA was randomly fragmented using the Covaris S2 Ultrasonicator system (Covaris, USA), producing DNA fragments primarily in the range of 200–300 nm. End-repair reactions were subsequently performed using T4 ligase, T4 alkaline phosphatase, and Klenow fragments. The repaired fragments were then ligated with sequencing adapters.

Standardized sequencing libraries meeting the requirements for Solexa sequencing were constructed. These libraries were amplified via polymerase chain reaction (PCR), and hybridization was performed on solid-phase chips manufactured by Agilent Technologies. Quality assessment of the hybridized libraries was conducted, and those meeting quality standards were subjected to genomic sequencing using the NovaSeq 6000 high-throughput sequencer (Illumina Inc., USA).

### 2.4 Data analysis

All sequencing data were aligned using BWA software (version 0.7.12-r1039), and the results were mapped to the human reference genome sequence (GRCh37/hg19). PCR products were processed using the PICARD tool (version 1.112) to remove duplicate sequences. Only sequencing data that passed quality control were included in the subsequent analyses.

The pipeline database platform was used for base calling, sequence assembly, alignment, and identification and annotation of single nucleotide polymorphisms and insertions/deletions (Indels). Candidate pathogenic variants were identified using ANNOVAR software. Frequently occurring variants were first filtered using established variant databases (e.g., dbSNP139, ESP6500, and 1000 Genomes Project). The remaining variants were annotated to specific genomic regions (e.g., exon, intron, 5′-UTR, 3′-UTR) to ensure accurate assignment to their corresponding gene segments.

Associations between known variants and clinical phenotypes were evaluated by analyzing variant types within protein-coding regions (e.g., missense, nonsense, or frameshift variants) using established medical databases, including OMIM, HGMD, and ClinVar. The pathogenicity and evolutionary conservation of the identified variants were assessed using two widely adopted prediction tools: SIFT and PolyPhen. Variants with potential disease relevance and their characteristic variant patterns were prioritized for interpretation through genetic analysis. Functional analysis of non-coding regions was performed using FunSeq software to identify potentially deleterious variants.

### 2.5 Sanger sequencing verification

PCR primers were designed to amplify potential variant sites, which were subsequently verified by Sanger sequencing. The corresponding gene loci in the patients’ family members were analyzed.

## 3 Results

### 3.1 Sequencing results

Genetic testing indicated that both individuals Ⅱ-1 and Ⅱ-3 carried the heterozygous missense variant c.1381G > A (p.Asp461Asn) in the *MYH7* gene. Pedigree verification confirmed the presence of this variant in individuals Ⅲ-1, Ⅲ-2, and Ⅲ-4.

### 3.2 Phenotypic differences among pedigree members

The phenotypic features observed in individual Ⅱ-1 included type 2 diabetic ketosis, coronary artery disease (silent myocardial ischemia), hypertension, previous cerebral infarction, mild obstructive sleep apnea–hypopnea syndrome, and apical LVNC.

Individual Ⅱ-3 was clinically diagnosed with hypertension, coronary artery disease (unstable angina), hyperlipidemia, type 2 diabetes, previous cerebral infarction, LVNC, and suspected right ventricular NCCM.

The phenotypic features of individual Ⅲ-4 included arrhythmia (ventricular premature contractions), blood-filled sinusoids in the apical segments of the posterior and lateral walls of the left ventricle, and increased trabeculation of the inferior, posterior, and lateral left ventricular walls, as observed on contrast-enhanced echocardiography.

## 4 Discussion

LVNC, also referred to as spongy myocardium, is a rare form of clinical cardiomyopathy first described in 1926. In 2006, the American Heart Association officially classified LVNC as a type of inherited cardiomyopathy within the category of primary cardiomyopathies. In 2008, the European Society of Cardiology categorized it as an “unclassified cardiomyopathy”.

The estimated prevalence of LVNC ranges from 0.014 to 0.3 % in the general population, with approximately 12–50 % of affected individuals demonstrating a familial genetic background. Significant genetic heterogeneity has been reported, with autosomal dominant inheritance identified as the most common mode. Epidemiological data indicate that the prevalence in males is two to three times higher than in females.

The pathogenesis of LVNC remains incompletely understood. Under normal embryologic development, the myocardial stroma progressively condenses into a compact structure, typically occurring between the fifth and sixth weeks of gestation. During this period, the trabecular meshwork transforms into a dense myocardial layer, and the intertrabecular recesses become compressed to form the coronary microcirculatory system. This compaction process generally proceeds from the epicardium to the endocardium and from the base to the apex of the heart. When this process is disrupted during early cardiac development, the myocardium may exhibit two distinct layers: an outer compacted layer and an inner noncompacted layer. The inner layer is characterized by prominent trabeculations and deep intertrabecular recesses that communicate with the left ventricular cavity [6].

The clinical manifestations of LVNC are typically categorized into three major groups: (1) cardiac dysfunction, most commonly presenting as heart failure due to impaired ventricular systolic function, with symptoms such as chest discomfort, reduced exercise tolerance, and dyspnea; (2) arrhythmias, such as premature ventricular contractions or atrial fibrillation, often accompanied by palpitations,The appearance of J waves on an electrocardiogram (ECG) indicates cardiac damage and serves as a warning indicator for malignant arrhythmias [7], 8]; and (3) thromboembolic complications, such as ischemic stroke. Additionally, some individuals may exhibit abnormal cardiac morphology detected by echocardiography or electrocardiogram (ECG) in the absence of corresponding clinical symptoms and left ventricular condition [9], [10], [11].

Among the twin brothers and their family members described in this study, the principal clinical features included dizziness, excessive somnolence, poor blood pressure control, impaired perfusion, coronary artery disease–related cardiac insufficiency, and arrhythmias (e.g., premature beats).

This report describes a pedigree of familial NCCM associated with the missense variant c.1381G > A (p.Asp461Asn) in the *MYH7* gene. Currently, this variant has been reported only in association with dilated cardiomyopathy [1]. The p.Asp461Asn substitution is located within the head domain of the *MYH7* gene (amino acid residues 1–778), a functionally critical region involved in myosin motor activity. Pathogenic variants within this domain have been associated with increased penetrance and a higher incidence of SCD.

The *β*-myosin heavy chain, encoded by the *MYH7* gene, is the most abundant protein in myocardial tissue. It serves as a fundamental structural and functional component of myosin and constitutes a major element of the thick filament in myofibrils. The protein comprises 1,935 amino acid residues and is structurally divided into head, neck, and tail domains. Of these, the head domain contains 778 residues at the N-terminus, the neck domain contains 289 residues, and the tail domain comprises 868 C-terminal residues, which form a coiled-coil *α*-helical structure. The cardiac *β*-myosin heavy chain plays a key role in myocardial contraction, with the head and neck domains mediating contractile regulation. The tail domain facilitates dimer formation via coiled-coil interactions, contributing to thick filament stability.

Prior studies have highlighted substantial interindividual variability in the clinical manifestations of LVNC. Therefore, investigation of genotype–phenotype correlations in LVNC is essential for early diagnosis, individualized treatment, and accurate prognostic assessment. To elucidate the clinical relevance of each variant site, the impact of gene variants on the biological function of the encoded protein must be assessed. The Asp461 variant described in this study is located in exon 14 of the *MYH7* gene. Exon 14 lies within the hinge region between two adjacent homocysteine residues in the globular head of the *β*-myosin heavy chain, a region critical for its functional activity.

Other reported pathogenic variants in the head domain of *MYH7* include Arg453Ser, Gly425Arg, Thr446Pro, Phe468Leu, and Thr441Met, which have been detected at a relatively high frequency in clinical studies. Research has shown that variants in this domain can enhance the ATPase activity of the myosin S1 sub fragment and may impair conformational dynamics or interactions with actin and other regulatory proteins, ultimately contributing to the pathogenesis of hypertrophic cardiomyopathy. However, to date, no variants in this domain have been linked to LVNC.

This study provides the first evidence of a potential association between head domain variants in the *MYH7* gene and LVNC. Comparative sequence analyses have demonstrated that the affected amino acid residues are highly conserved across species, suggesting that alterations in this region may significantly disrupt critical physiological functions. The Asp461Asn variant resides in the hinge region of the MYH7 head domain (exon 14), a critical locus for myosin motor activity. This substitution impairs conformational flexibility and ATPase kinetics, diminishing actin-myosin cross-bridge cycling efficiency. During embryogenesis, this energy-deficient state disrupts trabecular compaction, particularly the apex-to-base progression, creating the structural substrate for LVNC. Postnatally, metabolic stressors (e.g., diabetic ketoacidosis) and hemodynamic overload (e.g., hypertension) exacerbate contractile dysfunction, accelerating heart failure and arrhythmogenesis. This dual-hit mechanism – developmental compaction defect compounded by adult-onset energetic compromise – explains the incomplete penetrance and clinical heterogeneity. In summary, the Asp461 variant in the *MYH7* gene is considered a pathogenic variant with potential to cause syncope and SCD.

The marked phenotypic heterogeneity among family members harboring the MYH7 p.Asp461Asn variant underscores the complex interplay between genetic determinants and acquired modifiers in shaping LVNC expression. Located within the hinge region of the *β*-myosin heavy chain head domain (exon 14), this variant likely disrupts myosin conformational flexibility and ATPase kinetics, impairing actin-myosin cross-bridge cycling efficiency and precipitating an energy-deficient state that provides the structural substrate for noncompaction. However, divergent clinical manifestations demonstrate that comorbidities substantially modulate disease penetrance: in II-1, metabolic stress from diabetic ketoacidosis exacerbated myocardial energy depletion and electrical instability; II-3’s long-standing hypertension increased ventricular wall stress, promoting progressive dilation of the noncompacted layer while superimposed coronary artery disease created an “ischemic insult” that accelerated systolic deterioration; conversely, III-4, free from metabolic or vascular burden, exhibited isolated arrhythmia, suggesting that early phenotypes in younger carriers may predominantly involve electrophysiological perturbations. This three-dimensional “genetic-environmental-age” interaction model elucidates why identical pathogenic variants produce a spectrum dominated by heart failure, ischemia, or arrhythmia. Compared with previously reported MYH7 head-domain variants (e.g., Arg453Cys, Gly425Arg) primarily linked to hypertrophic or dilated cardiomyopathy, this study establishes the first association between Asp461Asn and LVNC, expanding the MYH7 cardiomyopathy spectrum beyond classic phenotypes. While HCM manifests concentric hypertrophy and DCM presents with global systolic dysfunction, our patients displayed prominent trabeculation with relatively preserved ejection fraction (48–61 %) yet higher thromboembolic risk, indicating that LVNC may represent a distinct pathobiological entity arising from a dual-hit mechanism: embryonic compaction arrest compounded by adult-onset contractile protein dysfunction. These findings mandate individualized, genotype-driven management. For carriers with metabolic syndrome (II-1, II-3), SGLT2 inhibitors should be prioritized to improve myocardial energetics, with early anticoagulation considered given CHA_2_DS_2_-VASc scores ≥2. III-4 requires cardiac magnetic resonance to quantify fibrosis burden and assess ICD candidacy despite preserved systolic function. All carriers warrant lifelong surveillance: adolescence focusing on arrhythmia screening, midlife monitoring metabolic comorbidities, and older age emphasizing thrombosis prevention. Ultimately, allele-specific RNA silencing or base editing targeting MYH7 represents a promising future direction to alter the natural history of this progressive cardiomyopathy.

LVNC is a rare form of cardiomyopathy with an incompletely understood pathogenesis. Significant challenges remain regarding its management and long-term prognosis. Clinical manifestations are non-specific, ranging from asymptomatic presentations to severe outcomes such as heart failure, arrhythmias, thromboembolism, or SCD. Studies have shown that early-onset LVNC is associated with more extensive and less compacted myocardial involvement, which may exacerbate clinical manifestations and adversely affect long-term outcomes.

Comprehensive TTE remains the most widely used diagnostic modality for LVNC. Currently, no disease-specific therapy is available. Management focuses on early identification and timely intervention to mitigate the risks of heart failure, SCD, and thromboembolic events. Early recognition and proactive management are essential to improving quality of life and prolonging survival. The management of LVNC patients follows approaches similar to those for other cardiomyopathies, including evidence-based heart failure treatment for LV systolic dysfunction, appropriate arrhythmia management, and consideration of oral anticoagulants to prevent thromboembolic events [12], 13]. Currently, there are no specific treatment recommendations for LVNC in the ESC guidelines. The management of LVNC-related complications follows established protocols for the underlying conditions. For heart failure with systolic dysfunction, standard care includes the use of SGLT2 inhibitors, and for end-stage heart failure refractory to optimal medical therapy, evaluation for left ventricular assist devices or heart transplantation is necessary. Tachycardiomyopathy Treated With Ablation by Using 3D Mapping System in a Patient With Friedreich Ataxia” illustrates how tailored intervention can significantly modify disease progression in rare cardiomyopathies. Patient II-1, with diabetes mellitus, presented with episodic sleepiness concerning for diabetic ketoacidosis; management involved stringent glycemic control and selection of antihyperglycemic agents with low ketoacidosis risk, which resulted in symptomatic improvement. Patient II-3, with hypertension and coronary artery disease, was managed according to contemporary guideline-directed medical therapy and revascularization strategies to mitigate major adverse cardiovascular events. Therefore, we propose a genotype-driven, phenotype-tailored management framework that stratifies patients into distinct risk categories. For asymptomatic young carriers (<30 years), management should encompass annual Holter monitoring with exercise testing and baseline cardiac MRI at diagnosis; while those harboring metabolic syndrome require prioritization of SGLT2 inhibitors to improve myocardial energetics coupled with early anticoagulation when CHA_2_DS_2_-VASc scores reach ≥2; and for carriers with significant ischemic burden, aggressive coronary revascularization must be pursued alongside consideration of ICD implantation even in the setting of borderline ejection fraction. Furthermore, all carriers mandate lifelong cascade screening. This stratified approach aligns with emerging evidence that gene-specific therapies are the future of rare cardiomyopathy management.

One limitation of this study is that the pathogenic mechanism of the MYH7 p.Asp461Asn variant has not yet been confirmed in functional experiments. Future studies, such as those utilizing induced pluripotent stem cell-derived cardiomyocyte models or gene-edited animal models, will help to elucidate its precise molecular pathophysiological role.

In the context of gene-targeted therapeutic strategies for LVNC, variants in the *MYH7* gene play a central role in disease pathogenesis. Detailed investigation of the pathological mechanisms associated with this genetic variant, along with the development of targeted therapeutic approaches, may yield important advances in improving patient outcomes and long-term prognosis.
